# Multidimensional nanomaterials for the control of stem cell fate

**DOI:** 10.1186/s40580-016-0083-9

**Published:** 2016-09-21

**Authors:** Sy-Tsong Dean Chueng, Letao Yang, Yixiao Zhang, Ki-Bum Lee

**Affiliations:** Department of Chemistry and Chemical Biology, Rutgers, The State University of New Jersey, Piscataway, NJ 08854 USA

**Keywords:** Nanoparticles, Graphene, Electrospun nanofibers, Biomaterials, Stem cells, Differentiation

## Abstract

Current stem cell therapy suffers low efficiency in giving rise to differentiated cell lineages, which can replace the original damaged cells. Nanomaterials, on the other hand, provide unique physical size, surface chemistry, conductivity, and topographical microenvironment to regulate stem cell differentiation through multidimensional approaches to facilitate gene delivery, cell–cell, and cell–ECM interactions. In this review, nanomaterials are demonstrated to work both alone and synergistically to guide selective stem cell differentiation. From three different nanotechnology families, three approaches are shown: (1) soluble microenvironmental factors; (2) insoluble physical microenvironment; and (3) nano-topographical features. As regenerative medicine is heavily invested in effective stem cell therapy, this review is inspired to generate discussions in the potential clinical applications of multi-dimensional nanomaterials.

## Background

A major drawback in current stem cell therapy is the limited control over stem cell fate, which leads to low efficiency in giving rise to mature differentiated cells that can replace the original damaged cells [1, 2]. On the other hand, ex vivo differentiation of stem cells have been proven to be very low in efficiency and has poor cell survival upon transplantation into the body. To overcome these challenges, various multidimensional nanomaterials that are capable of precisely controlling stem cell fate in the nanometer range have been developed rapidly. Furthermore, nanomaterials are highly versatile in nature, they enable us to effectively and dynamically control the differentiation of stem cells solely through the biophysical cues of nanomaterial [3]. As demonstrated, subtle changes in the physical microenvironment such as the surface material orientation, ECM protein composition, and shape can significantly influence the therapeutic potential of stem cell [4].

**Fig. 1:**
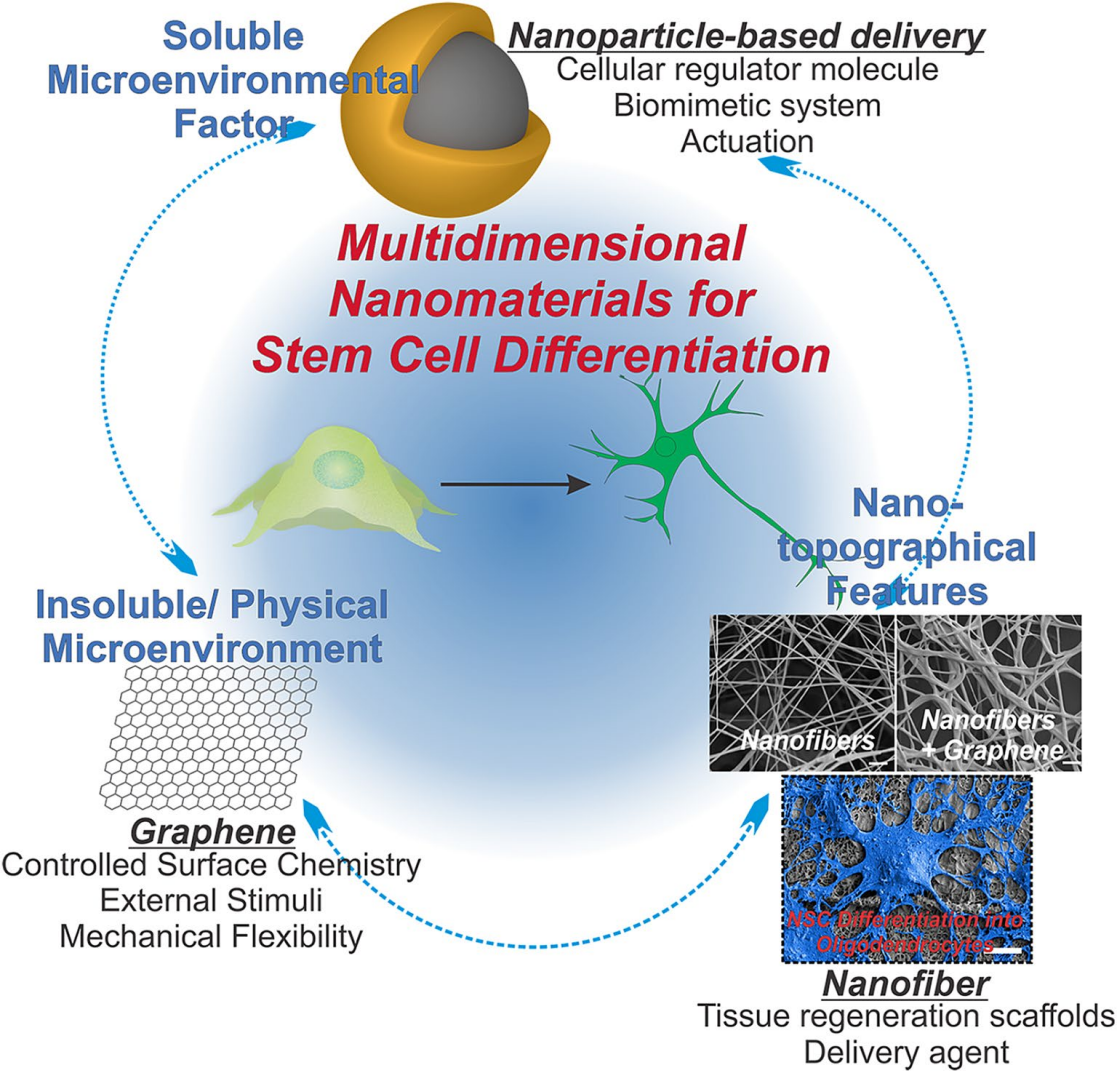
Illustrative diagram representing the multidimensional nanomaterials discussed in this review article: soluble microenvironmental factors, insoluble physical microenvironment, and Nano-topographical features

This review covers novel nanomaterials used for stem cell differentiation in multidimensional approaches. Nanotechnology-based approaches to selectively guide stem-cell-based regeneration include: (1) soluble microenvironmental factors; (2) insoluble physical microenvironment; and (3) Nano-topographical features (Fig. 1). Soluble microenvironment describes the growth factors, cytokines, and chemokines associated with nanomaterials delivered to the stem cells. Insoluble physical microenvironment describes the biochemical cues given to extracellular matrix (ECM) protein for enhanced attachment and orientation. Lastly, nano-topographical feature describes the physical and topographical cues nanomaterial provides to the stem cell. Overall, nanotechnology-based approaches offer physicochemical control required to differentiate stem cells into cell lines of interest. With the increasing interest to develop innovative tools and technologies, we can also expect creative solutions for the complex problems associated with stem cell biology and their applications.

## Diffusive microenvironmental factor

With their unique sizes in the range of viruses and proteins, nanomaterials can interact with biological systems at the molecular level with high specificity [5]. Nanoparticles, different bulk materials, possess significant surface to volume ratio, composition, shape, surface, and unique optical and/or magnetic properties that are advantageous in solving biomedical challenges. Apart from numerous biomedical applications like imaging and drug/gene delivery, application of directing stem cell differentiation through nanoparticles is lacking. However, the unique properties of nanoparticles are met with strong enthusiasms from researchers for modulating stem cell behaviors and understanding stem cell signaling mechanisms [6].

### Cellular regulator molecules delivery

Regulator molecules including growth factors and signaling molecules are major factors with the key ability to regulate stem cell behaviors, However, naturally occurring regulator molecules suffer from short circulation half-life and fast degradation rate under in vivo circumstances. These drawbacks together with low diffusivity render the real application of stem cell therapy inefficient due to the ineffective delivery and non-specific distribution. As such, a delivery system with spatial–temporal precision is of significance for utilizing signaling molecules to guide stem cell differentiation. With high surface-to-volume ratio, high loading capacity and targeting delivery modality, nanoparticles have frequently been used as signaling molecule carriers. Owning to their intrinsic properties, nanomaterials can provide prolonged growth factor releasing profile to treat stem cells effectively above concentration threshold. For example, hepatocyte growth factor (HGF) was loaded into chitosan nanoparticles (CNPs), formed by an ionotropic gelation method through strong electrostatic interactions between the CNPs and proteins, to show the successful steady release of 85 % HGF for 5 weeks. As shown in in vitro differentiation experiments, the treated mesenchymal stem cells (MSCs) adapted to a round-shape hepatic cell characteristic morphology with upregulated expression of albumin [7]. Further in vivo study was done by co-injection of MSCs with HGF-CNPs into cirrhotic mice [8]. The in vivo differentiation of MSCs of hepatocytes was confirmed by the expression of albumin and cytokeratin 19. The increased level of alpha-fetoprotein and decreased expression of alpha-smooth muscle actin and type-I collagen suggested the reversal of fibrosis of hepatic extracellular matrix.

Inorganic nanoparticles, especially nanoporous/mesoporous silica nanomaterials have been used as biomolecule carrier for stem cell differentiation in bone tissue regeneration. Neumann et al. coupled Bone morphogenetic protein 2 (BMP2) on nanoporous silica nanoparticle through amino-silane linker to test the osteoinductive effect on adipose-derived human mesenchymal stem cells (ADMSCs) [9]. Apart from osteogenesis, Kolzova and colleague used nanoporous silica particles to deliver exogenous trophic mimetics Cintrofin and Gliafin, peptide mimetics for the ciliary and glial cell derived neurotrophic factors, to embryonic stem cells (ESCs). Confirmed by immunostaining, the embryonic stem cells were driven into motor neurons with the delivery of two peptide mimics. The function of the differentiated motor neuron was also characterized through electrophysiology and voltage-sensitive fluorescent protein imaging. Furthermore, the differentiated motor neurons were transplanted into mice, showing long-term survival, demonstrating the potential application in ESC differentiation for stem cell therapy [10].

By incorporating small molecules into polyelectrolyte nanoparticles consist of polyethyleneimine (PEI) and dextran sulfate (DS), Santos et al. delivered retinoic acid into the subventricular zone (SVZ) to induce neural stem cells differentiation [11]. The differentiated neuronal function was assessed through intracellular calcium variations upon KCL depolarization and histamine stimulation. Additionally, nanoparticle-based genetic manipulation has also been shown as an alternative strategy to guide stem differentiation.

The versatility of nanoparticles also allows target delivery of genetic molecules into the cells. Lee and coworkers have firstly demonstrated that using magnetic core–shell nanoparticles (MCNPs) to guide neural stem cells (NSCs) differentiate into different lineages (neurons and oligodendrocytes) with the delivery of genetic materials of small interfering RNA (siRNA) or plasmid DNA. The controlled differentiation of neural stem cell was succeeded in RNA interference-based approach by suppressing two key “neural switch” genes CAVEOLIN-1 and SOX9 for oligodendrocyte and neuron differentiation respectively [12]. Chen et al. also demonstrated the hepatic differentiation of induced pluripotent stem cells (iPSCs) using mesoporous silica nanoparticles (MSNs) as a non-viral gene carrier and cell imaging agent. The mesoporous silica nanoparticle based carrier showed minimal cytotoxicity and fast cellular uptake for iPSCs. Upon treatment of MSNs loaded with hepatocyte nuclear factor 3β(HNF3β) plasmid DNA, the iPSCs went into mature hepatocyte lineage differentiation with functions like low-density lipoprotein uptake and glycogen storage [13].

As shown, small molecule dosing and genetic manipulation are equally significant for directing stem cell fate in tissue engineering and regenerative medicine [14]. With this merit, Lee et al. demonstrated the co-delivery of small molecules and RNA interference agents to differentiate neural stem cells into neurons using a single vehicle delivery system based on the cyclodextrin-modified dendritic polyamine. Through the binding of small molecule retinoic acid with β-cyclodextrin and electrostatic interaction between siRNA and dendritic polyamine, the combination of small molecule and RNA interference synergistically targeted multiple cellular pathways to induce stem cell differentiation. The controlled and reliable neuronal differentiation was confirmed through immunostaining of GFAP and TuJ1 markers [15].

### Nanomaterial biomimetic system

**Fig. 2:**
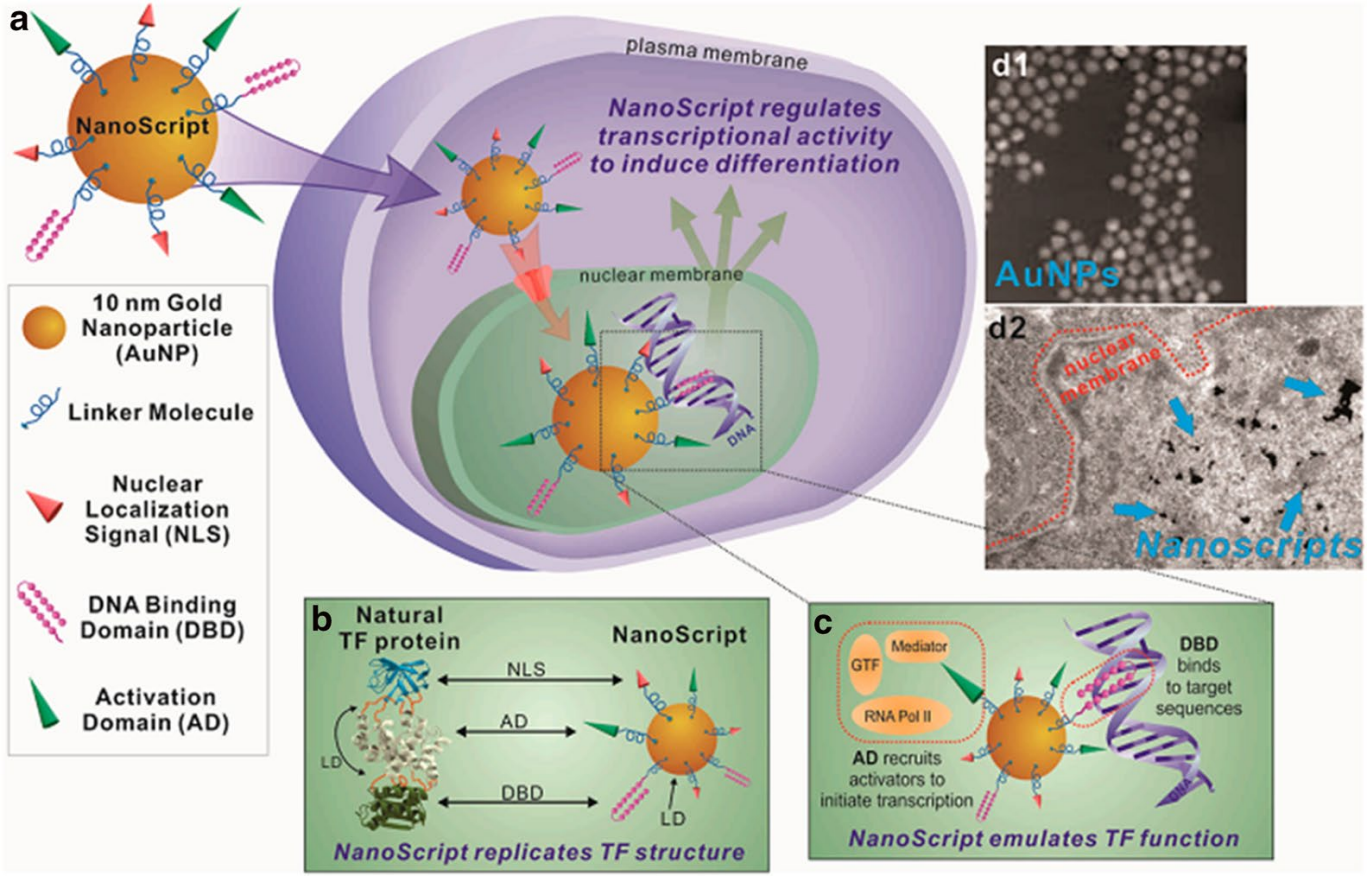
General design scheme of NanoScript. **a** NanoScript is consist of a single 10 nm gold nanoparticle, DNA binding domain (DBD), activation domain (AD), and nuclear localization signal (NLS). The assembly of all components mimics a natural transcription factor. **b** Comparison of NanoScript with a natural transcription factor. **c** The DBD and AD domains on NanoScript work synergistically to mimic natural transcription factors for transcriptional modulation on expression of targeted genes. **d1** and **d2** NanoScript shows high mono-dispersity, efficient uptake, and nuclear localization [18]

Transcription factors are master regulators in orchestrating basic cellular behaviors and are responsible for critical cellular functions and cellular fate. Therefore, by modulating the expression of specific genes, the differentiation of stem cell can also be modulated through manipulating the key transcription factors [16, 17]. Contrary to traditional viral-based delivery systems with drawbacks such as cytotoxicity, immunogenicity, and undesirable for clinical applications, Lee has developed NanoScript, a **nano**particle-based synthetic tran**script**ion (Fig. 2) [18]. Specifically, NanoScript consists of (1) a nanoparticle core, usually gold nanoparticle due to its biocompatibility and ease of functionalization; (2) functional peptides for nuclear localization; (3) an activation domain mimic; and (4) a Py-Im hairpin polyamide as synthetic DNA binding domain. To demonstrate stem cell differentiation, NanoScript was designed to mimic myogenic regulatory factors (MRFs), which are a group of four transcription factors, MyoD, myogenin, Myf5, and Mrf4, functioning as a crucial regulator of muscle cell differentiation. The NanoScript-MRF successfully guided ADMSCs to differentiate into mature muscle cells showing upregulated myogenin and myosin expression and myofibrils formation [19].

Furthermore, with the ability to activate endogenous gene expression activity, NanoScript was conjugated with* N*-(4-chloro-3-(trifluoromethyl)phenyl)-2-ethoxybenzamide (CTB) derivative, an epigenetic modulator, to enhance chondrogenic differentiation from adipose-derived mesenchymal stem cells. Specifically, the CTB derivatives conjugated on NanoScript, triggering the p300 signaling pathway as a histone acetyltransferase (HAT) activator will induce an increase in HAT activity, transforming the chromatin structure from “tight” into “loose” form. One gene that is regulated by the p300 signaling pathway is *Sox9,* a key chondrogenic promoting gene. Thus, the combination of CTB derivatives and Sox9 activation, NanoScript showed enhanced chondrogenic differentiation from ADMSCs [20].

In addition to previously mentioned advantages, NanoScript can be flexibly functionalized with interchangeable components to mimic different transcription factors as well. Once natural transcription factors bind to their target genes, they can activate or repress gene transcriptions. Contrasting gene activation using the NanoScript platform, a gene repressing NanoScript was made to emulate the repression ability of natural transcription factor to downregulate gene expression at the transcription level in late 2015. By designing the repression NanoScript to downregulate Sox9 expression, neural stem cells were successfully differentiated into neurons. The mature neuron function, calcium ion flux, was observed [21].

### Actuating nanoparticles

**Fig. 3:**
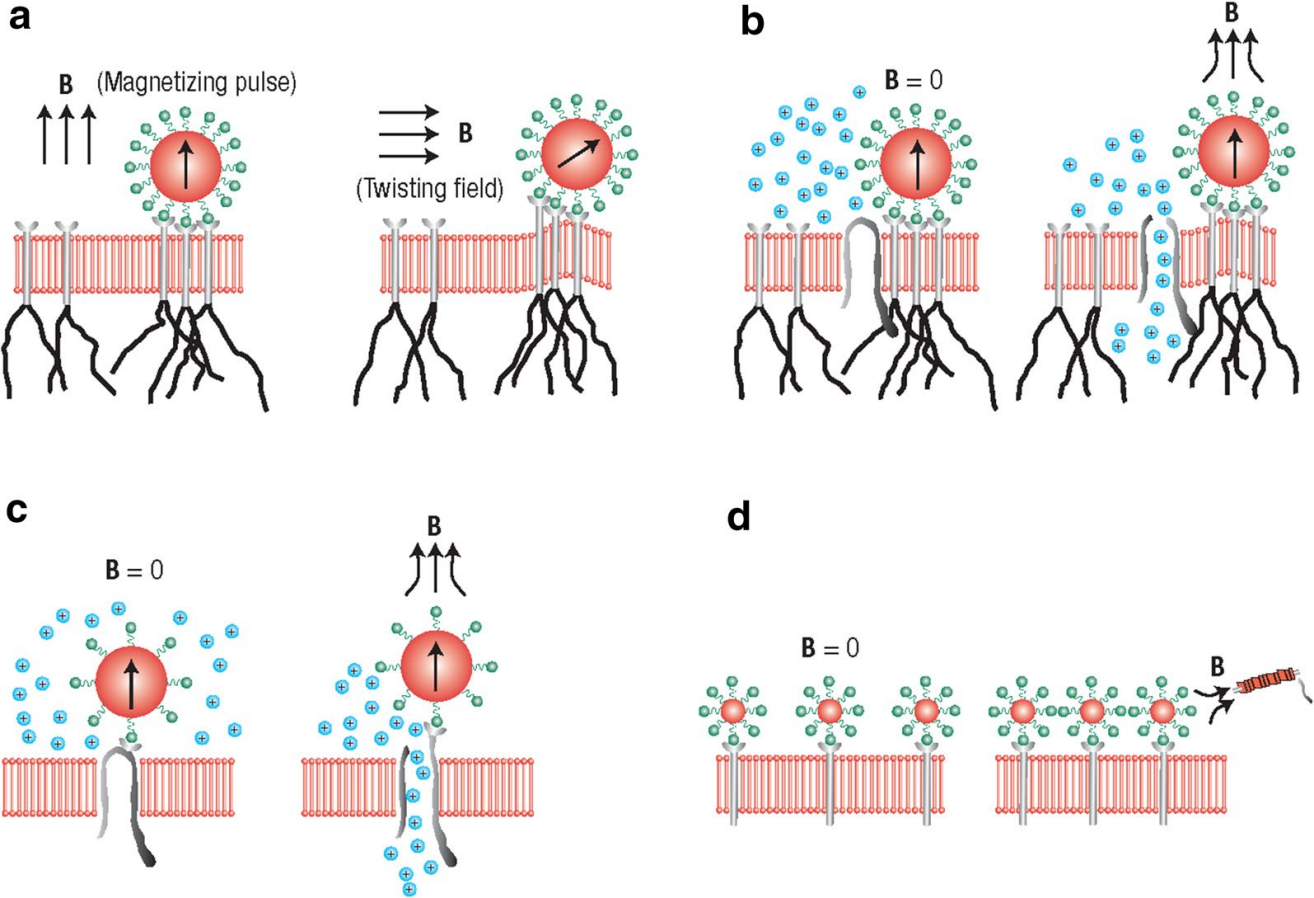
Different types of magnetic actuation. **a** Magnetic twisting cytometry; **b** mechano-sensitive ion-channel activation; **c** targeted ion-channel activation; and **d** receptor clustering [26]

Other than providing soluble cues, nanoparticles have also been shown to provide mechanical cues responsible for stem cell fate determination, tissue formation, and organ regeneration. Recently, remote magnetic actuation (Fig. 3) has been demonstrated to provide mechanical stimulation to biological cells [22]. Upon mechanical stimulations, the integrin receptors at the focal adhesion of cells have been shown to correlate with cell biochemistry, morphology, and even epigenetic chromosomal activity [23, 24]. With the development of magnetic nanoparticles, cellular or even receptor level magnetic actuation can be achieved to activate different mechanosensors existing in the cell membrane [25]. Through facile surface functionalization, nano-actuators can bind to the cell surfaces and manipulate cell function or even guide stem cell differentiation with external magnetic field. Magneto actuation technology offers a method to isolate single receptor-mediated cellular mechanotransduction process which can bring insights to related cellular–matrix interactions [26].

Among the very first demonstrations of this approach, Ingber and his colleagues attached magnetic nano/microbeads to cell-surface through integrin receptors with applied tensional forces. The cellular responses were recorded with different kinds of mechanic stimuli: pulse, oscillation, static stress, and prolong stress. Through the cellular adaption to the mechanotransduction, several pathways related mechanisms like Rho signaling and mechanosensitive ion channels were identified to be responsible for the different adoption for static and dynamic mechanical changes applied to integrin [27]. Later, the similar magnetic nanoparticle-based approach was applied to generate a mechanical stress to specific ion channel of interest (i.e. TREK-1). The study demonstrated the specific activation of a mechanosensitive ion channel in real time through force generated on targeting nanoparticle on the extracellular region of TREK-1 [28]. More recently, magnetic nanoparticles have been utilized to generate magneto-mechanical stimulation on cell surface receptors for stem cell differentiation. Henstock et al. targeted the same receptor mentioned above, TREK-1, with the delivery of 4pN per nanoparticle for mechanotransduction in mesenchymal stem cells, resulting in a 2.4-fold increase in the mineralization in the chick fetal femur [29]. Furthermore, due to facile functionalization on the magnetic nano-actuators, different mechano-sensitive receptors can be modulated simultaneously to study receptor interactions and pathway interplays. Hu et al. demonstrated higher mineralization ratio with the help of osteogenic culture medium and stimulating two specific cell membrane receptors: platelet-derived growth factor receptor α (PDGFRα) and integrin ανβ [30]. Another example of this combined receptor mechanical stimulation was demonstrated by Haj and his colleague by targeting PDGFRα and PDGFRβ. Upon cyclical magneto-mechanical stimulation, human bone marrow-derived mesenchymal stem cells (hBMSCs) differentiated into a smooth muscle cell lineage [31]. Overall, the unique size range and properties of nanoparticles enable nanoparticle-based stem cell regulatory approach with molecular level specificity, improved interaction efficiency, and spatial–temporal resolution. A nanoparticle-based stem cell differentiation system with the ability to interact with cellular processes and deliver regulatory molecules remotely on demand would be of significance for translating the current research to the next stage. Moreover, development of such nanomaterials with desirable degradability would be a key step for the advancement in clinical applications of nanoparticle-based stem cell therapy and tissue engineering.

## Insoluble physical microenvironment

During stem cell differentiation, cells exert forces to and simultaneously receive forces from the surrounding extracellular matrix (ECM) proteins. Therefore, mechanical properties from the ECM play a significant role in regulating stem cell behaviors. Moreover, the physical stimulations (e.g. electrical, mechanical, and photochemical stimulation) from the substrate can provide an additional dimension of control over the differentiation process of stem cells. Furthermore, the physical microenvironments of the ECM also influence the clinical transplantation potential of stem cells. To this end, a variety of organic and inorganic scaffolds, insoluble physical microenvironments, that can mimic the ECM have been developed to have precise control over stiffness, surface topography, shear forces, degradability, and retractability. Among the various types of nanomaterials, tremendous interest has been focused on two-dimensional structured nanomaterials in the last decade since the discovery of graphene—a sp [2] bonded carbon nanomaterial [32, 33]. A variety of graphene derivatives and graphene mimics have been rapidly designed, synthesized, and studied. In 2008, graphene was reported as a drug delivery vehicle for the first time, and generated intense interest in graphene-based bioapplications, ranging from biosensing, cancer therapy, drug delivery, and regenerative therapy [20, 34]. For stem cell culturing and differentiation, graphene and its derivatives have been found universally to be versatile, biocompatible, and highly stable scaffolds for promoting stem cell differentiations with low inflammatory induction [35]. The broad interest generated from graphene nanosheet-based scaffolds have further inspired the development of scaffolds based on other two-dimensional nanomaterials such as ultrathin polymeric nanosheets, which is biocompatible and biodegradable. For example, the high mechanical flexibility would allow sufficient tolerance of mechanical stresses for tissue regeneration. Also, the highly absorptive and porous architecture of 2D nanomaterial constructed scaffold would be advantageous for efficient mass transport. Moreover, the high electrical conductivity of graphene-based scaffold allows electrical stimulation, monitoring, and detection of differentiated neurons or cardiomyocytes. With high mechanical flexibility and versatile surface functionalities, graphene and their derivatives can be facilely engineered into scaffolds with tunable geometrical and mechanical cues to direct stem cell fate and further enhance stem cell differentiation.

### Enhancing stem cell differentiation through substrate surface chemistry

**Fig. 4:**
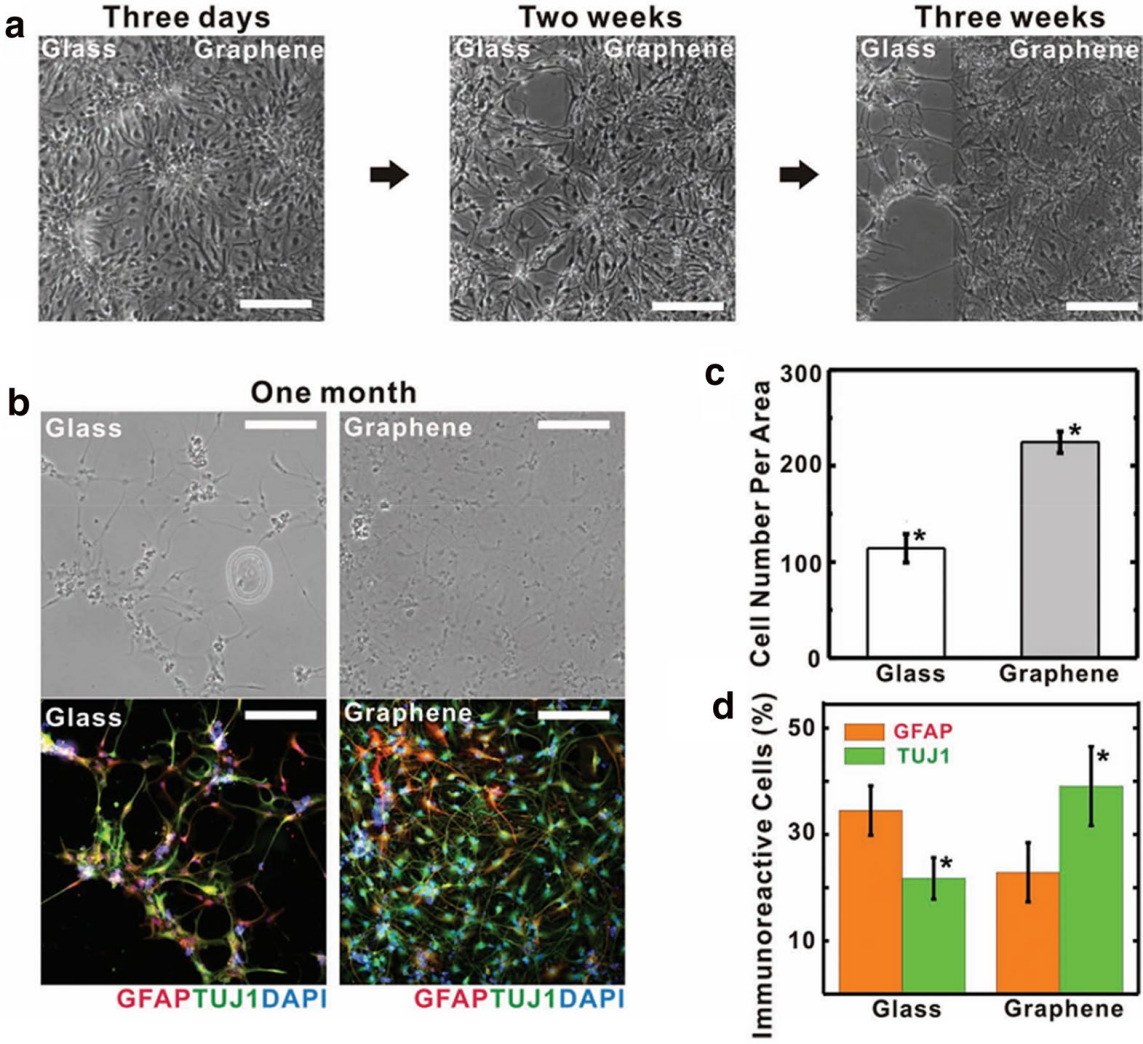
Enhanced neural differentiation of hNSCs on CVD grown graphene substrate. **a** Bright-field images of the hNSCs after a differentiation process of 3 days (*left*), 2 weeks (*middle*) and 3 weeks (*right*). **b** Bright field (*top*) and fluorescence (*bottom*) images of hNSCs after differentiation on glass (*left*) and graphene (*right*) after a differentiation process of 1 month. Immunostaining on GFAP (*red*) and TUJ1 (*green*) for astroglial and neural cells were conducted on hNSCs. **c** Cell density (per 0.64 mm^2^) on graphene substrate and glass after 1-month differentiation. **d** Percentage of GFAP (*red*) and TUJ1 (*green*) on glass and graphene. All *scale bars* are 200 μm [36]

Graphene has been demonstrated as a biocompatible and promising substrate for electrical and optical interfacing devices due to their high mechanical flexibility, transparency, and conductivity. Hong et al. reported that graphene substrate fabricated by chemical vapor deposition (CVD) effectively enhanced the differentiation of human neural stem cells into neurons (Fig. 4) [36]. While the mechanism remains unclear, laminin-related cellular pathways were found to be significantly enhanced and the graphene substrates were observed to act as an excellent cell-adhesion layer especially for the long-term differentiation process. Later on, also using a CVD method, Cheng et al. found that mouse hippocampal neurons cultured on graphene showed enhanced neurite sprouting and outgrowth, which could act through the GAP43 related pathways [37]. As the cell adhesion and growth factor is highly related to the surface functional groups of graphene, fluorinated graphene sheets have been developed as a scaffold to guide neural stem cell growth and differentiation as well. Loh et al. observed a further enhancement of neuronal differentiation from MSCs after they introduced neuron-inductive agent, retinoic acid, which could be attributed to an enhanced absorption and binding of retinoic acid towards the fluorinated substrate [38]. Similar to the observations in neurogenesis, enhanced cellular adhesion and proliferation on scaffolds constructed from graphene and its derivatives have also been found in the osteogenic, myogenic, chondrogenic, cardiomyogenic, and other differentiation processes in MSCs [19, 39–42].

While the mechanism is still unclear, hydrophilicity, surface functionality, roughness, surface area, and nanotopographical features such as ripples were proposed to be the reasons for such enhanced adhesion. Loh et al. reported the chemical roles of graphene and graphene oxide (GO) in guiding stem cells towards specific cell lineages. They suggested that the strong noncovalent binding towards osteogenic inducers of graphene make it act as a preconcentration platform for enhanced osteogenesis [43]. They also found that differentiation into adipocytes was suppressed on graphene-based scaffolds as insulin, a key adipogenic growth factor, was denatured through the π–π interaction on graphene scaffold. GO, on the other hand, did not interfere with the adipogenesis because they bind with insulin through electrostatic interaction. For chondrogenic differentiation, Lim et al. fabricated a cell-assembled graphene 3D biocomposite and showed enhanced chondrogenic differentiation [19]. Kim et al. later discovered that GO plays a dual role, both as an excellent cell-adhesion substrate but also as a growth factor protein preconcentration platform during the chondrogenic differentiation process [41]. In contrast to the conventional chondrogenic pellet culturing and differentiation of MSCs, the incorporation of GO preloaded with transforming growth factor-β3 (TGF-β3) can overcome the diffusion limitation of TGF-β3 that occurs inside the pellet. Chondrogenic marker, SOX-9, and Aggrecan expression were enhanced more than twofold and threefold respectively compared to the control group. Among the different types of stem cell differentiation, preliminary investigation on the graphene-based scaffolds for osteogenesis and neurogenesis has been conducted in vivo, confirming their high biocompatibility and promising applications in tissue engineering.

### Electrical and optical stimulation for enhanced stem cell differentiation and detection

**Fig. 5:**
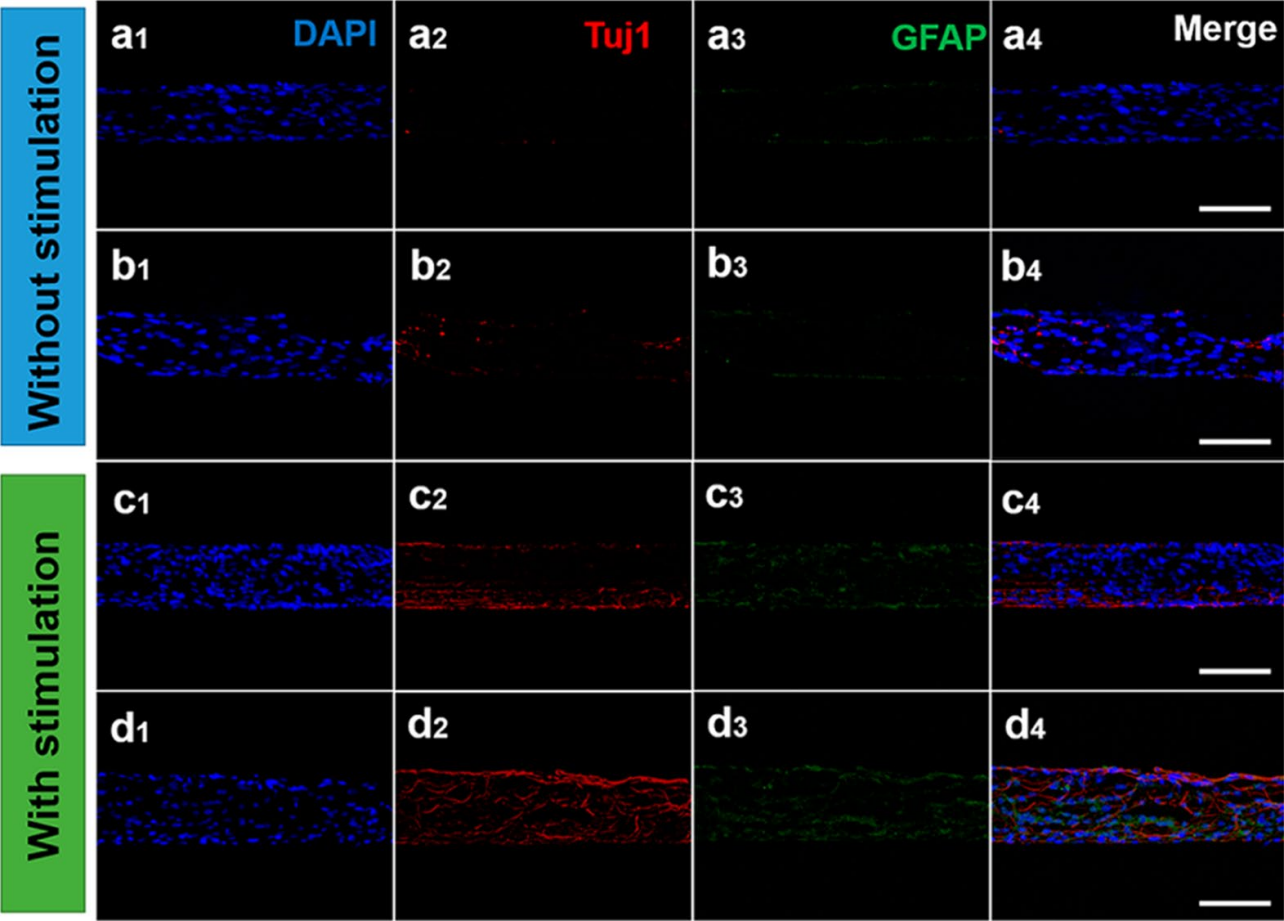
**a**, **b** After cultured under stimulation and without electrical stimulation for 21 days, cells were immunostained with DAPI (*blue*) for nucleus, Tuj1 (*red*) and GFAP (*green*). **c**, **d** After cultured under TENG electrical stimulation and without TENG electrical stimulation for 21 days. All *scale bars* are 100 μm [44]

In addition to its surface chemistry and high aspect ratio, graphene also has unique optical and electrical properties that can stimulate stem cells and further assist differentiation. Pulse electrical stimulation has been proven to enhance the neuronal regeneration efficiently. However, it would be more practical to integrate a power supply inside the body instead of inserting electrodes. Recently, to address this challenge, Wang et al. constructed a self-powered electrical stimulation system (high effective triboelectric nanogenerator, TENG) that utilized a graphene-based hybrid microfiber to enhance the differentiation of neural stem cells through electrical stimulation (Fig. 5) [44].

Another study has also successfully utilized electrical field stimulation to control neural cell–cell interactions through alternating the protein synthesis related to cell mobility and cytoskeleton. More importantly, the graphene substrate also provides a good electrical coupling with the neurons for electrical stimulations. Ghaderi et al. demonstrated the differentiation of human neural stem cells (hNSCs) into neurons using reduced graphene oxide (rGO); while under pulsed laser stimulation, the photothermal effect induced radial thermal flow and resulted in the organization of the neuronal network by elongating the differentiated neurons in the radial directions [45]. In contrast, unreduced graphene oxide (GO), where there is a weaker photothermal effect or quartz, no obvious enhanced differentiation was observed. The same group has also reported the photo-catalytical stimulation on hNSCs by utilizing an rGO/TiO_2_ hybrid scaffold [46]. They found that the flash photostimulation not only promoted proliferation (by a factor of ~2.5) of the stem cells, but also guided stem cells differentiation into neuronal lineage versus glial cells. Recently, the stimulation of cardiomyocytes differentiated from ADSCs has also been demonstrated on the graphene scaffold [42].

In addition to stimulating and enhancing differentiation of stem cells, the excellent electrical and optical properties have also been utilized for detecting the behaviors of differentiated cells and for monitoring the differentiation process. For example, it is reported that the neural network can be successfully formed on graphene films and the neural signals can be effectively enhanced on graphene films [37]. Graphene can act as a conductive substrate and transfer the electrical signals to the neural cells cultured and efficiently modulate neural cell behaviors. Furthermore, Choi et al. also synthesized a scaffold assembled from GO encapsulated gold nanoparticles (Au@GO NPs) that is applicable for monitoring the differentiation of NSCs based on electrochemical detection and surface-enhanced Raman Spectroscopy (SERS) [47]. It has been reported that during the stem cell differentiation, C=C bonds gradually decrease, which can be reflected from the Raman bands at 1656 cm^−1^ [1]. Based on this mechanism, Au@GO NPs monitored the differentiation in a non-destructive manner. By taking advantage of electrical properties of graphene, electrochemical detection of the C=C bonds was also achieved in a single platform.

### High flexibility for enhanced differentiation and facilitated transplantation

**Fig. 6:**
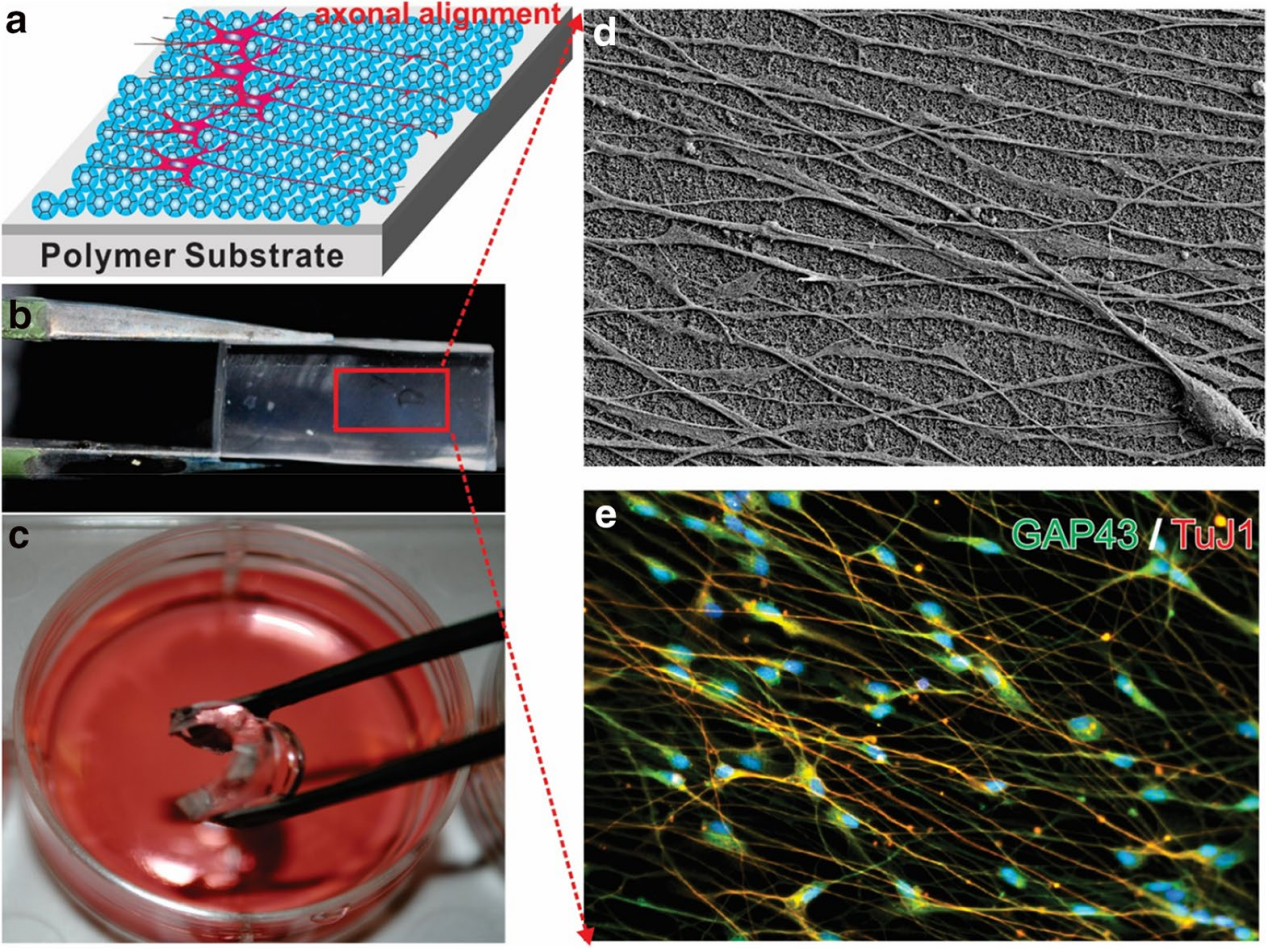
Axonal alignment of differentiated neurons cultured on flexible and biocompatible SiNP-GO thin film on polydimethylsiloxane (PDMS) substrate. **a** Schematic diagram of axonal alignment of differentiated neurons on multidimensional nanomaterial substrates. **b** SiNP-GO monolayer on PDMS. **c** Flexible nanomaterial substrate suitable for cell culture. **d** FE-SEM image of highly aligned axons from Day 14 culture on SiNP-GO PDMS substrate. **e** Immunocytochemistry results showing neuronal marker TuJ1 and axonal marker GAP43 expression [48]

When acting as a coating material, the high flexibility of graphene can effectively take on the geometry, pattern, and morphology of the underlying scaffolds. Lee et al. demonstrated a silica nanoparticle-graphene oxide hybrid scaffold to promote axonal alignment of differentiated neurons (Fig. 6) [48]. Recently, Lee also developed micro-contact printing technique and fabricated combinatorial patterns of GO for the effective control over the differentiation of human adipose-derived mesenchymal stem cells [3] (ADMSCs). The morphology of ADMSCs was effectively modulated by the GO patterns. It was found that ADMSCs preferentially differentiate into osteoblasts and the grid pattern selectively guides the ADMSCs into neuronal lineage with highly elongated axons. Not only the aforementioned 2D scaffolds, 3D scaffolds based on graphene and its derivatives have also been fabricated by taking advantage of their high flexibility. These 3D scaffolds could accelerate the application of graphene for tissue engineering due to the recent interest in 3D cell culture in the biological field. For example, based on layer-by-layer (LBL) assembly, Shin et al. reported a GO-embedded GelMA hybrid hydrogel scaffold that forms multiple layers of cardiomyocyte cell sheet [49]. The high flexibility of GO was proposed to facilitate cell separation and stack for the highly dense, organized 3D complex tissue architectures. Most importantly, this tissue-like cell construct demonstrated synchronous and spontaneous beating after 24-h culture process.

Recently, scientists have fabricated a variety of 3D nanostructures based on GO and graphene using hydrothermal, electrostatic assembly, and soft templating methods. Liu et al. reported a three-dimensional hydroxyapatite–graphene hybrid forms assembled from graphene for enhanced osteogenesis [39]. Moreover, the mineralized 3D scaffold further accelerated and enhanced osteogenesis of MSCs through the increased deposition of inorganic minerals. In addition to graphene-based nanosheets, polymeric nanosheets also present high level of flexibility, which is highly advantageous for performing transplantation and adapting to local injured areas. Recently, Fujie et al. reportedly inserted magnetic nanoparticles embedded PGA nanosheets scaffold along with in vitro cultured monolayer retinal pigment epithelial cell (RPE) for retinal recovery [50]. Due to its ultrathin nature and flexibility, the polymer nanosheet scaffold was proposed to avoid vitreous fluid leakage and minimize postsurgical infection.

Overall, the unique surface chemistry, binding toward biomolecules, fascinating electrical and optical properties of graphene-based nanosheets, and the excellent mechanical flexibilities of 2D nanomaterial have demonstrated high biocompatibility, enhanced cellular adhesion, proliferation, stem cell differentiation, detection, and transplantations. Future research would call for further investigations, especially in simulation studies on the mechanism on how graphene binds to bio-molecules and how graphene enhances and accelerate the differentiation process. Furthermore, development of novel 2D nanomaterials assembled in 3D scaffold with biodegradable properties and studying the stem cells in vivo would further boost the clinical application of 2D nanomaterials in tissue engineering.

## Nano-topographical features

In addition to soluble cues, stem cells are very sensitive to the surrounding physical and topographical microenvironment as well. The act of modifying the underlying substrates allows researchers to control and regulate cell adhesion, spreading, shape, elongation, and ultimately cell fate [51]. As tissue formation is heavily dependent on the recruitment of progenitor cells from the surrounding area, biomaterials introduced as implants are critical in bridging the gap when the defects are too severe to heal autogenously. Therefore, it is important for biomaterials to be able to orchestrate the biochemical and biophysical cues to facilitate cell–cell and cell–ECM interactions to facilitate stem cell therapy.

### Nanofiber based stem cell differentiation scaffold

Nanofiber technology has gained significant attentions and excitement in the research and development field as a potential solution to overcome some of the current challenges such as burn and wound care, tissue and organ regeneration, and various degenerative diseases in biomedical engineering. Compared to traditional bulk materials, nanofiber substrates offer tremendous amount of surface area for enhanced cell adhesion, protein adhesion, and drug loading. Furthermore, nanofibers offer topographical features mimicking the macrophysical structure of natural ECM proteins in both animals and humans. Lastly, nanofibers can be fabricated through various processes and materials which have the industrial potential to be regulated and scaled up easily for mass production [52].

Typically, a nanofiber can be generated through various methods including molecular self-assembly, electrospinning, and thermally induced phase separation [53]. With rational material design, through the use of biodegradable polymer, a nanofiber can provide time-dependent temporary support until the regenerated tissue is matured. Through the introduction of the interconnected porous network, nanofibers have also been shown to promote cell–cell interaction through deep cell penetration. Additionally, a nanofiber can be fabricated by ECM protein to promote stem cell adhesion and differentiation. Moreover, a nanofiber can be controlled through fabrication to either be random or aligned to give anisotropic topographical guidance. Furthermore, bioactive compounds (growth factors, nucleic acids, and integrin-binding ligands) have also been shown to be embedded into nanofiber scaffolds. To realize the potential of nanofiber biomedical application, numerous works have been focused on the topic of tissue regeneration.

### Skin tissue regeneration

During the early stages of nanofiber technologies in biomedical applications, many natural polymers were used. For example, in skin tissue regeneration, Park and Min et al. had demonstrated through type I collagen nanofiber [54] and surface modification of silk fibroin (SF) nanofibers with oxygen gas to increase surface hydrophilicity [55], they were able to promote the cellular activity of human dermal keratinocytes and fibroblasts. Nie group [56] and Sethuraman group [57] had used a different blend of biodegradable chitosan materials to form nanofibers that are comparable in tensile strength of normal human skin to evaluate for skin regeneration in vitro. By attaching bone-marrow-derived mesenchymal stem cells (BM-MSCs), Ma group demonstrated that by increasing the density of BM-MSCs, thanks to the biomimetic nanofiber scaffolds, wounds treated with BM-MSCs attached nanofibers closed more than a week earlier than untreated controls [58].

### Bone regeneration

Through a co-self-assembling peptide of phosphorylated serine peptide amphiphile and RGDS peptide amphiphile, Stupp and co-workers inserted the peptides based nanofiber into a 5 mm rat femoral critical-size defect to demonstrate bone formation and mineralization within 4 weeks [59]. To form the self-assembling nanofiber, the nanofiber-forming molecules contain a peptide segment with one domain that has a strong propensity to form extended β-sheets and the second domain with residues for bioactivity. The β-sheets domain is crucial for promoting assembly of fibrous aggregate instead of spherical aggregate [60]. By combining synthetic biodegradable polymers, Ramakrishna was able to increase the porosity of polycaprolactone/hyaluronic acid/gelatin to over 93 % and maintain tensile strength to support osteoblast for mineralization [61]. This interconnecting porous composite nanofibrous scaffold provided large surface area for cell attachment, cell activity, and cell proliferation. Similar to the previous study [58], MSCs have also been cultured on to completely synthetic polycaprolactone nanofiber to show deep penetration of cells and the presence of abundant ECM after 1 week [62]. In the same report, Vaccanti group also showed that the cultured MSCs on the surface of PCL nanofibers were inclined to differentiate into osteogenic lineages as mineralization had occurred after 4 weeks [62].

### Ligament regeneration

Nanofibers have also been applied to ligament regeneration. Unlike other tissues, tendon and ligament have a very low propensity to regenerate due to their high ECM density and low vascularity [63]. The body typically relies on scar tissue mediated healing process which is inadequate to replace the functions of damaged or diseased tendon and ligament. As mentioned, owning to its high porosity nature, nanofiber allows for high cell infiltration rate and also allow for uniaxial alignment to mimic the anisotropic structure of native tendon and ligament. In this report [64], Ouyan and coworkers demonstrated that by seeding human tendon progenitor cells (hTSPCs) on top of aligned Poly(lactic acid) (PLLA) nanofibers higher tendon gene expression similar to native tissue was observed compare to randomly aligned fiber control which is significantly lower. The reason is that ECM production of tendon and ligament fibroblasts have specific uniaxial direction. In another approach [65], Shin stretched the nanofiber at 12 cycle/min frequency for 24 h and found that human ligament fibroblast-produced more ECM collagen on longitudinally stretch axis than the transverse axis.

### Hepatocytes

Typical nanofibers have sizes above 400 nm in diameters. However, through rational design, by functionalizing chitosan nanofiber with galactose to make galactosylated chitosan (GC) and shrinking the nanofiber to ~160 nm, Gu and coworkers showed the enhanced bioactivity and mechanical stability of primary hepatocytes through mimicking the ECM properties of hepatocytes [66]. Through topographical properties of nanofibers, Baharvand [67] was able to enhance the generation of hepatocyte-like cells from mesenchymal stem cells with commercially available Ultra-Web™ nanofiber with the help of inducing bio-agents. From his finding, hepatocyte markers ALB, CYP7a1, and HNF4α were consistently upregulated compared to regular tissue culture condition.

### Neural tissues

The brain has long been considered to be more complex than the universe, and yet this spectacular piece of “organic machinery” has fascinated the scientists and clinicians endlessly. When there are subtle disturbances to the brain, complications in physical, motor, psychological, and cognitive functions can occur. Therefore, the understanding of how the central nervous system (CNS) functions and developing therapies to repair this intricate system after damages caused by diseases and injuries has been longed-for by the scientists and clinicians. To differentiate into specialized neural cells of interest (e.g. neurons and oligodendrocytes), researchers have been exploring the 3D microenvironment for gradient diffusion of bio-agents, cell migration, and cell–cell interaction. Zhang and coworkers [68] have developed a 3D culture system by attaching several functional motifs to self-assembling peptide RADA16. Comparing to recombinant ECM proteins, peptide-based nanofiber offers not only topographical bio-mimic but also the high in purity and amount of desired functional motifs. In the region with higher biological motifs, neural cells are significantly enhanced in survival. Similarly, by presenting neurite-promoting IKVAV motif through 3D self-assembled peptide nanofiber, Stupp group had also shown his artificial nanofiber scaffold can rapidly induce neuronal differentiation from neural progenitor cells [69].

**Fig. 7:**
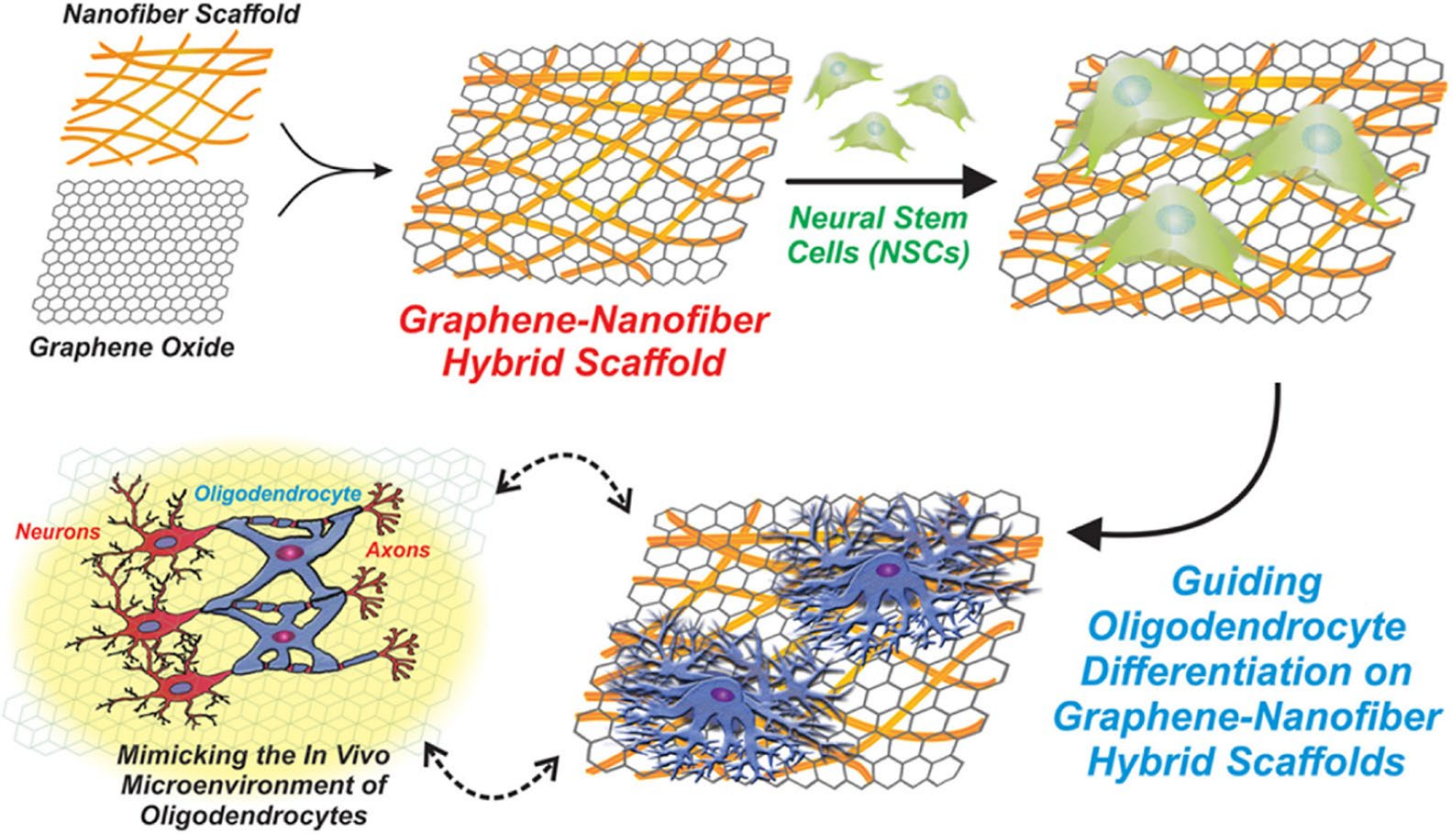
Schematic diagram depicting the fabrication and application of graphene-nanofiber hybrid scaffolds. Polymeric nanofibers generated using electrospinning were subsequently coated with graphene oxide (GO) and seeded with neural stem cells (NSCs). NSCs cultured on the graphene-nanofiber hybrid scaffolds show enhanced differentiation into oligodendrocyte lineage cells [70]

**Fig. 8:**
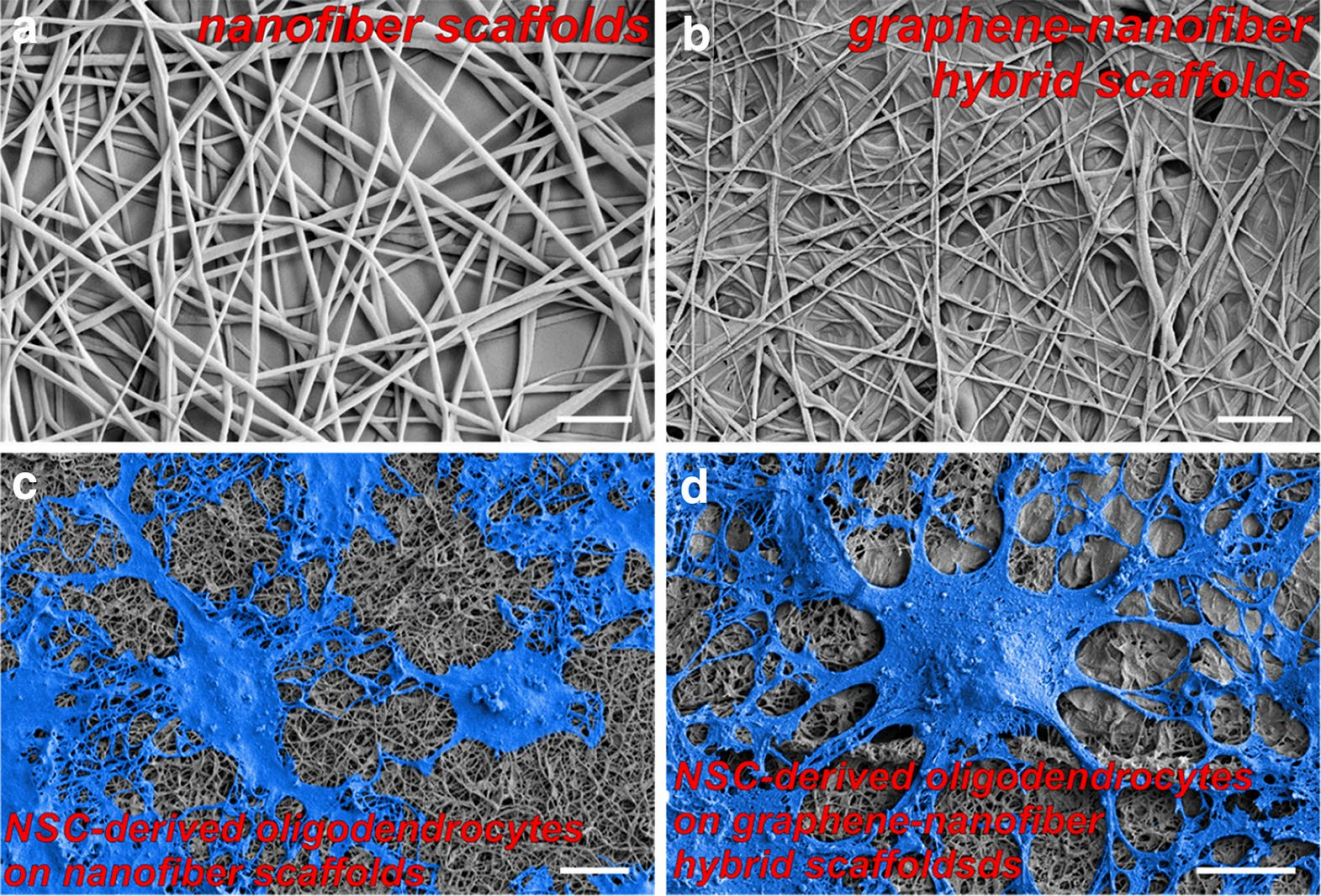
Morphology of scaffolds and cultured NSCs on plain scaffold and graphene oxide (GO) coated nanomaterial-nanofiber hybrid scaffold. Field emission scanning electron microscopy (FE-SEM) images of PCL nanofibers (**a**) and PCL nanofibers coated with 1.0 mg/mL GO solution (**b**). *Scale bars* 2 µm. FE-SEM images of oligodendrocytes derived from neural stem cells (NSCs) after 6-days culture on PCL nanofibers (**c**) and nanomaterial-nanofibers hybrid scaffold (**d**). Cells are pseudo-colored blue for contrast. Process extension of NSC-derived oligodendrocytes is clearly exhibited when cultured on the nanomaterial-nanofiber hybrid scaffold (**d**) over PCL nanofiber only control (**c**). *Scale bars* 10 µm [70]

**Fig. 9:**
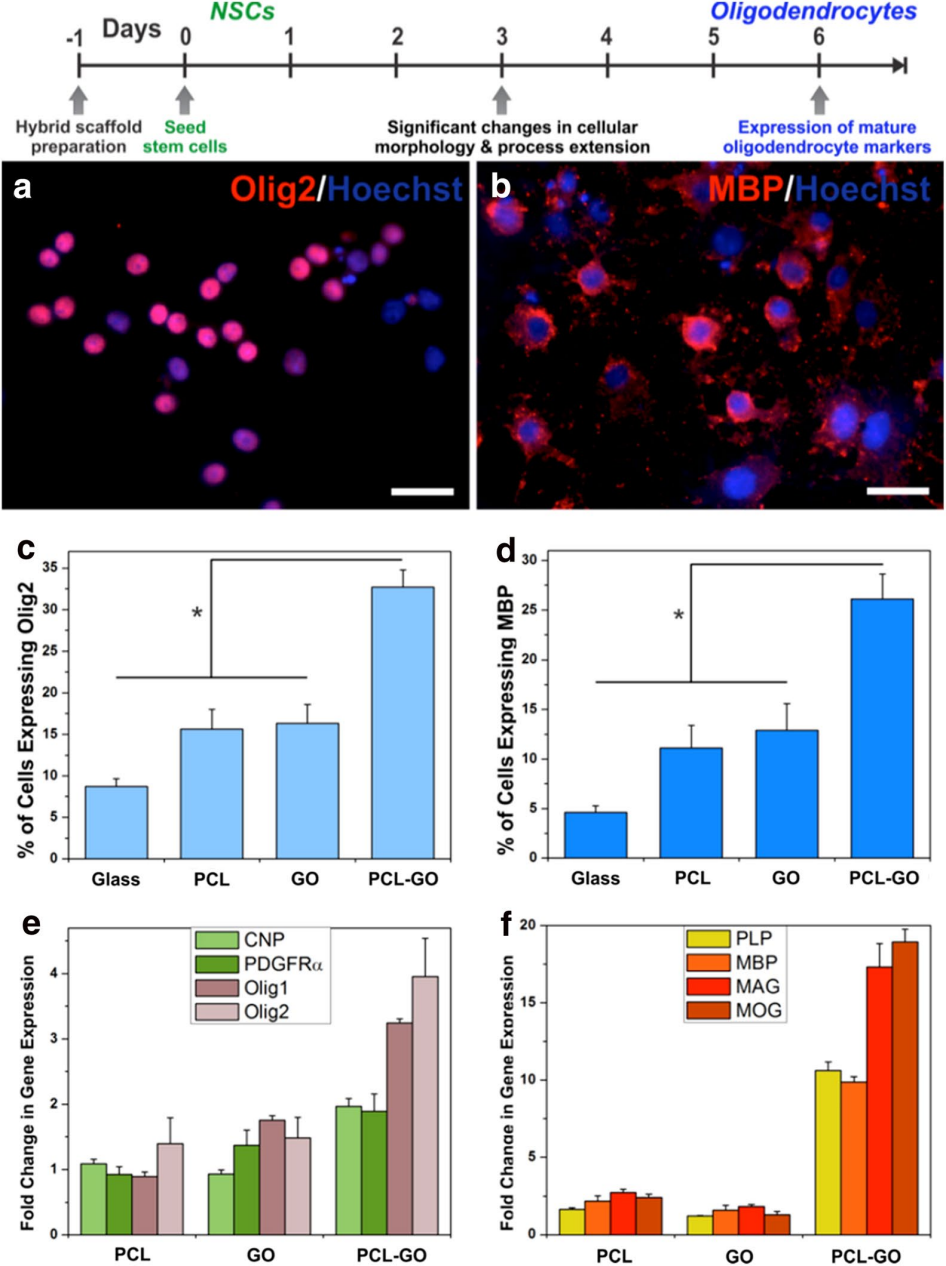
Enhanced oligodendrocyte differentiation on PCL-GO nanofiber-nanomaterial hybrid scaffold. Immunocytochemistry image of NSCs after 6 days of culture on hybrid scaffolds, stained for the early oligodendrocyte marker Olig2 (**a**) and the mature oligodendrocyte marker MBP (**b**). *Scale bars* 20 µm. Quantitative comparison on various substrates of the percentage of cells expressing Olig2 (**c**) and MBP (**d**). Quantitative PCR analysis showing gene expression of early oligodendrocyte markers including CNP, PDGFR, Olig2 and Olig2 (**e**), and mature oligodendrocyte markers including PLP, MBP, MAG and MOG (**f**). The gene expression is relative to GAPDH, and normalized to the conventional PLL-coated glass control [70]

For the CNS regeneration, a number of studies have been focused on the differentiation of neurons, while oligodendrocyte—a myelinating cell lineage involved in many neuronal circuits, was underappreciated. In combination with two-dimensional nanomaterial, Lee group reported a polycaprolactone (PCL)—GO hybrid scaffold for guiding stem cell differentiation into oligodendrocytes (Fig. 7) [70]. The scaffolds were fabricated from electrospinning of nanofiber scaffolds, followed by drop-casting GO solutions. The nanofiber morphology, which is a mimic of oligodendrocyte ECMs, was found to be well maintained after GO drop-casting, and the GO provides an excellent surface for cell adhesion and differentiation (Fig. 8). From the polymer chain reaction (PCR) analysis, while the PCL only and GO only (control groups) only has one to three-fold enhancement of oligodendrocyte markers compared to the control group (glass), the PCL-GO hybrid scaffold enhanced the oligodendrocyte differentiation by over 10 folds (Fig. 9). We have also proposed that such effective control over oligodendrocyte differentiation and development originate from integrin-mediated pathways, mainly FAK, Akt, ILK and Fyn.

### Nanofiber-based delivery of bioactive agents

To turn nanofibers into drug carries, bioactive agents are typically immobilized into the polymer matrix for their control release. Depending on the polymer material, typical procedure consists of entrapment [71] or binding [72] as demonstrated by Stupp et al. By entrapping the bioactive agents in an intermediate state, bioactive agents are physically encapsulated inside of the cross-linked polymers. Another method of loading bioactive agents into nanofiber is to bind the bioactive agents chemically onto the polymer structure of nanofiber through hydrogen bonds, covalent bonds, hydrophobic and electrostatic interactions.

Drug release from nanofibers can be described through three mechanisms: desorption from fiber surface, diffusion through fibers, and in vivo fiber degradation [73]. When the nanofiber carrier is subjected to a physiological condition, body fluid or tissue culture media will penetrate the space in between individual nanofibers. When the nanofiber drug carrier is swollen by the aqueous phase, drugs or proteins attached to the fiber surfaces can be released. Upon desorption from fiber surface, drugs will be disused into the aqueous phase.

## Conclusion and future perspective

Stem cell therapy holds the key to regenerative medicine for functional recovery from various injuries and diseases. Addressing the current challenges, nano-chemists and biologists have invested in various nanomaterials and their assembly in multidimensional domains to mimic the properties of the natural microenvironment to promote and dictate stem cell differentiation into desired lineages. In this review, the benefits of nanomaterial in the field of stem cell biology are clearly shown to be advantageous over traditional methods including bio-reagent delivery, in vivo imaging modality, and transplantation platform. Although much has been investigated to this point, there remains more investigation to be done in the clinical applications of multi-dimensional nanomaterials.
